# Humanistic and economic aspects of haemophilia treatment in Bulgaria. Comparison between two therapeutic approaches: prophylactic vs. on-demand treatment

**DOI:** 10.1080/13102818.2014.926687

**Published:** 2014-08-26

**Authors:** Guenka Petrova, Konstantin Tachkov, Svetla Georgieva, Maria Dimitrova

**Affiliations:** ^a^ Faculty of Pharmacy, Medical University of Sofia, Sofia, Bulgaria; ^b^ Medical University of Sofia, University Hospital ‘Alexandrovska’, Sofia, Bulgaria

**Keywords:** haemophilia, cost-effectiveness, prophylactic therapeutic regime, on-demand regime

## Abstract

The aim of the present study was to calculate the cost-effectiveness of on-demand and prophylactic treatments of severe haemophilia A for Bulgarian patients. The point of view is that of all patients suffering from severe haemophilia A. An epidemiological model was created, which includes data regarding the number of patients divided into age groups up to 74 years. In the model, the transition age from prophylactic to on-demand treatment was gradually increased to up to 40 years. Costs of blood clotting factor, hospitalization, major surgery and indirect costs were considered; incremental cost-effectiveness ratio was calculated. The results showed that despite the increase in the costs for factor VIII with 20 million BGN, the saving obtained from other health services and indirect expenses reduce the overall expenses with 5.3 million BGN. If there is a gradual increase in the age when patients are transferred from a prophylactic to an on-demand regimen, the costs for factor VIII would increase from 10.4 million to 19.7 million BGN, but due to a decrease in indirect costs as well as other health service costs, the total costs would decrease. The sensitivity analysis showed that the costs for clotting factor VIII are what influences the cost-effectiveness in both regimes. This indicates that decreases in the factor VIII cost will increase the overall efficiency in both regimes. In conclusion, the application of the prophylactic regime for patients up to 40 years of age will provide better treatment, increase the quality of life and decrease the incremental costs.

## Introduction

The medical and economic benefits for treatment of severe haemophilia A through the application of a prophylactic therapeutic regime or an on-demand regime is a topic greatly discussed by many authors; even so, there is still no unanimous consensus on the matter of which approach is more efficient and to what point it is cost-effective to use healthcare funds to cover the prophylactic approach.[1–5]

The therapeutic advantages of the prophylactic regime are indisputable.[6–8] It ensures that patients have the opportunity of developing normally, especially patients in their childhood.[9–12] Of economic significance are the decrease in the frequency of bleeding, the decrease in the number of joint haemorrhages, decrease in days of hospitalization and decrease in the days of inability to work.[3,4,11,13] The humanistic characteristics of the prophylactic regime are measured by the quality of life of patients.[14,15]

Neither of the two therapeutic regimes has been economically assessed and evaluated in terms of costs and results in Bulgaria. This fact drew our interest towards carrying out this economic study.

The present study aims to calculate the cost-effectiveness of both on-demand and prophylactic treatments of severe haemophilia A for Bulgarian patients. The point of view is that of all patients suffering from severe haemophilia A.

## Materials and methods

### Description of the model

An epidemiological model was created, which includes data regarding the number of patients divided into age groups up to the age of 74 years (base model). The number of patients was gathered from national consultants. The calculations for the costs and the results in the base model for both therapeutic regimes – on-demand and prophylactic treatment, were made under the assumption that all patients are treated either with the one or the other regime.

Based on this base case model, a second model was developed in which the prophylactic treatment was applied only until the patient reached 18 years of age, after which they were transferred to an on-demand therapeutic approach. This is the current medical practice in Bulgaria. The second model was varied by gradually increasing the transition age to up to 40 years of age.

### Costs in the model

The costs for clotting factor VIII as well as other healthcare services were calculated and compared for all presented models. Only the cost for plasma derived coagulation factor was considered.

The model included the following costs: costs for the application of the therapeutic regimen (costs which are covered by the National Health Insurance Fund (NHIF) in the form of one international unit (IU) of factor VIII). The costs were calculated by multiplying the expected number of cases in each age group by the therapeutic dose per kilogram then multiplied by the cost of one IU factor VIII. The prophylactic therapeutic model suggests that the expected number of cases will take 20 IU/kg of factor VIII three times per week, whereas the dose under the on-demand treatment is 40 IU/kg administered when bleeding arises.

The hospitalization costs, in cases where there is a haemorrhage, were calculated by multiplying the expected number of yearly hospitalizations by the price of hospitalization (NHIF tariff cost); in addition the cost of factor VIII in both regimens was included. The costs of surgical interventions were calculated by multiplying the number of expected interventions by the price of an intervention (NHIF tariff cost), as well as separately adding the costs of factor VIII for both treatments.

The expected number of hospitalizations and surgical interventions was arrived at by multiplying the probability of their occurrence (based on literature referenced) in a respective age group by the number of cases in that same age group.

Indirect costs were calculated according to the human capital approach. The number of expected days out of work was multiplied by the average daily wage for the year 2013 (data by the National Statistical Institute).

All costs are based on prices from the year 2013 and are presented in national currency (BGN). The exchange rate is 1 euro = 1.958 BGN.

### Cost–utility analysis

The therapeutic result is displayed as quality adjusted life years (QALY) in both therapeutic regimes, where the quality of life is a constant for treatment regimens and is not influenced by changes in age.

The data for expected hospitalizations, days out of work, expected surgical interventions and quality of life were obtained from the referenced literature.

Total costs were calculated for both regimes and the respective different models were also divided by the changes of QALY, thus deriving the incremental cost–utility ratio.

### Sensitivity analysis

The sensitivity analysis was carried out by building a tornado diagram. The impact of the variables added to the model was evaluated, where the costs for factor VIII, cost for hospitalizations, for surgical interventions were varied within an interval of ±30% and their respective influence on the incremental cost-effectiveness ratios between costs and QALYs was also analysed.

## Results and discussion

The initial input data in the model are shown in Table 1. The costs of services are identical for both the prophylactic and the on-demand regimen; the differences are in the lower amount of haemorrhages, hospitalizations and days out of work, which are in favour of the prophylactic regimen.

**Table 1.** Input model parameters.

Parameter	Prophylactic regimen	‘On demand’ regimen
Number of patients (data from NHIF)	254	254
Dose regimen	20 IU/kg/3 times weekly	40 IU/kg/on demand (30 IU to 40 IU)
Number of haemorrhages yearly		20.91 (17–20.91)
Number of hospitalizations yearly	5 (1–6)	20.91 (17–33.2)
Number of days of inability to work	1 (1–3)	19 (9–19)
Yearly probability for surgical intervention	0.0008	0.0023
QALY	0.88 (0.88–0.92)	0.72 (0.68–0.74)
Cost of IU factor VIII (BGN)	0.60	0.60
Cost of surgery when bleeding (BGN)	700	700
Cost of hospitalization when bleeding (BGN)	570	570
Cost of extra factor VІІІ 5000 UI per surgery	0.60	0.60
Average daily wage (BGN)	34	34

**Figure 1.**
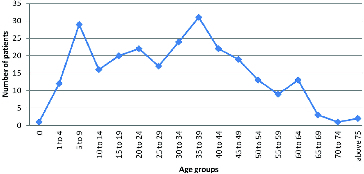
Number of patients with severe haemophilia in age groups.

The distribution of the number of patients in age groups is shown in Figure 1. The age group of over 70 contains only 3 people out of all 254 haemophilia A patients who are being treated to date.

### Base model

**Figure 2.**
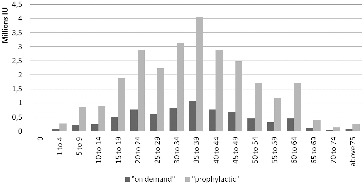
Necessary UI blood factor VIII in both therapeutic regimes.

The results obtained from the base model, i.e. under the assumption that all patients are treated either according to a prophylactic or to an on-demand regime only, showed that the required quantity of factor VIII is higher under the prophylactic regimen, due to its frequent application (Figure 2).

The costs for both therapeutic regimens increase in age groups, where for the prophylactic treatment the costs are higher, especially after the age of 9, due to the increase in patient weight, which requires an increase in the dose, as well as the sheer number of patients in the age groups of 15–60 years (Figure 2). The expenses in the on-demand regimen were calculated with a constant amount of haemorrhages (20.91 per year), although some authors point out that with age there is an increase in the amount of haemorrhages to 33 per year or more.[1,10]

The expenses for major surgical interventions when following the prophylactic treatment are threefold lower in comparison to those with on-demand treatment, and the hospitalization costs are fivefold lower.

The lower cost for hospitalizations and surgical interventions partly offset the costs for factor VIII and decrease the overall costs for the healthcare system.

The indirect costs, under which category falls the inability of a patient to work, are also lower with the prophylactic regimen. These expenses, despite not being covered by the NHIF, have a massive impact on patients, due to their decrease in income.

**Figure 3.**
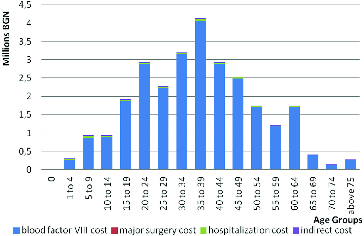
Total cost for prophylactic therapeutic regime.

**Figure 4.**
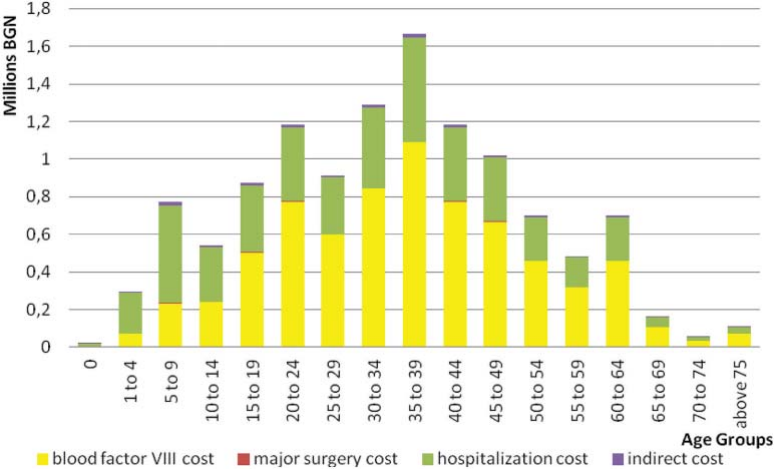
Total cost for on-demand therapeutic regime.

If the structure of total costs in both regimens is compared, it becomes apparent that with the on-demand treatment hospitalization expenses have the highest percentage, whereas with the prophylactic regimen the expenses for factor VIII are the highest (Figures 3 and 4).

The results obtained from the base case model clearly showed that, despite the increase in the costs for factor VIII with 20 million BGN, the saving obtained from other health services and indirect costs reduce the total costs with 5.3 million BGN (Table 2).

**Table 2.** Total costs (BGN) in the base case model where all patients are either on prophylactic or on-demand regime.

	Variables in base case dosage regime	Costs	Difference
Costs for factor VIII	Prophylaxis	27,878,572.80	20,404,971
	On demand	7,473,602.02	
Costs for surgical interventions	Prophylaxis	751.84	−1410
	On demand	2161.54	
Costs for hospitalization	Prophylaxis	1,612,900	−5,132,248
	On demand	6,745,147.8	
Indirect costs	Prophylaxis	8636	−155,448
	On demand	164,084	

### Transition between dosage regimes after the age of 18

Due to economic reasons not all haemophilia A patients can afford the prophylactic regime throughout their entire life. This is why some healthcare systems introduce restrictions when using this therapeutic regime, such as the case in Bulgaria, where patients are treated with the prophylactic regimen only until the age of 18, after which they are shifted over to on-demand treatment. This model showed that the extra expenses for factor VIII are reduced by 3 million BGN, but this is accompanied by a decrease in the savings from surgical interventions, hospitalizations and inability to work with about 1.2 million BGN when compared to the base model (Table 3).

**Table 3.** Total costs in the base case model where all patients are either on prophylactic or on demand regime.

	Variables in the current therapeutic practice	Costs	Difference
Costs for factor VIII	Prophylaxis	27,878,573	17,443,050
	Prophylaxis at the age of 18 then on-demand	10,435,523	
Costs for surgical interventions	Prophylaxis	752	−976.80
	Prophylaxis at the age of 18 then on-demand	1729	
Costs for hospitalization	Prophylaxis	1,612,900	−3,556,203
	Prophylaxis at the age of 18 then on-demand	5,169,103	
Indirect costs	Prophylaxis	8636	−107,712
	Prophylaxis at the age of 18 then on-demand	116,348	

**Figure 5.**
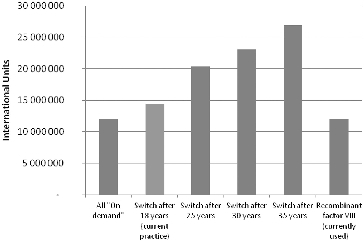
The necessary IU of factor VIII for prophylactic dosage regime in different age groups.

**Figure 6.**
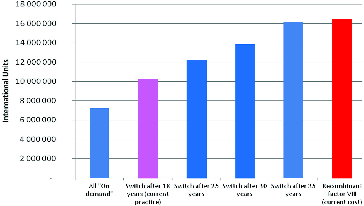
Cost of factor VIII for prophylactic dosage regime in different age groups.

These results question the effectiveness of this particular treatment approach, which is why we analysed how the costs for factor VIII would change when there is a gradual increase in the age when patients transition to on-demand treatment. Figure 5 shows the changes in the required quantity of factor VIII in IU under gradual transition from a prophylactic to an on-demand regimen, whereas Figure 6 shows the changes in expenses for Factor VIII.

In addition, the quantities and the monetary value of the recombinant factor VIII, which is used at the moment for the therapy of haemophilia patients, are also compared in the last column of Figures 5 and 6.

If there is a gradual increase in the age when patients are transferred from a prophylactic to an on-demand regimen, the costs for factor VIII increase from 10.4 million to 19.7 million BGN, but due to a decrease in indirect costs as well as other health service costs, this increase is partly offset (Table 4).

**Table 4.** Change in costs with increase of age when patients change to ‘on demand’ treatment.

	Cost factor VIII	Cost major surgery	Cost of hospitalization	Indirect cost
Switch 18	10,435,523	1728.64	5,169,103	116,348
Switch 25	12,615,904	1606.54	4,724,578	102,884
Switch 30	14,300,744	1512.19	4,381,081	92,480
Switch 35	16,679,342	1378.99	3,896,144	77,792
Switch 40	19,751,697	1206.94	3,269,767	58,820

### Cost–utility analysis

An incremental analysis of overall total costs and acquired benefits in treatment with the different regimes was carried out. With the increase of patients’ age, when the prophylactic regime is used, there is also an increase in the additional costs for a unit QALY from 63,000 to 485,000 BGN per patient. It was found that the highest expenses are observed in the situation where all haemophilic patients are on the prophylactic regime (Table 5) – 1.8 million BGN for additional QALY.

**Table 5.** Incremental cost–utility ratio.

	Total cost	Incremental cost	Total QALY	Incremental QALYs	Incremental cost–utility ratio (ICUR)
All on demand	11,959,712		183		65,354
Switch at 18	15,722,703	3,762,991	196	13	289,461
Switch at 25	17,444,973	1,722,270	199	3	574,090
Switch at 30	18,775,817	1,330,844	202	3	443,615
Switch at 35	20,654,657	1,878,840	205	3	626,280
Switch at 40	23,081,491	2,426,834	210	5	485,367
All on prophylactic	44,976,098	21,894,607	224	12	1,824,551

### Sensitivity analysis

**Figure 7.**
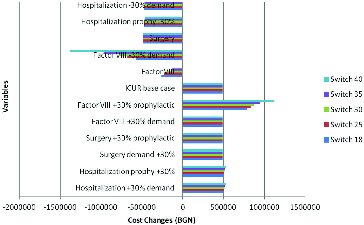
Tornado diagram for sensitivity analysis.

The sensitivity analysis showed that the costs for clotting factor VIII are what influence the cost-effectiveness of the alternatives in both regimes – on-demand and prophylactic. This indicates that decreases in the cost of factor VIII would increase the overall efficiency in both regimes (Figure 7).

### Final remarks

This study is the first of its kind in Bulgaria and raises questions which the Bulgarian therapeutic health insurance is unable to answer. The practice of applying prophylaxis in haemophilia A patients up to 18 years is not cost effective, but neither is the on-demand therapy for all severe haemophiliacs if we apply the threshold of three times the gross domestic product (GDP) per year. This requires a thorough re-evaluation of the practice of providing prophylaxis for patients up to 18 years of age, as the loss of quality of life is significant.[16]

The implementation of a prophylactic regime for all 254 patients being treated for type A haemophilia will increase anti-clotting medicines cost, but will decrease the cost for hospitalization and surgical intervention. The patient community will benefit more in accepting a prophylactic regime as the main form of treatment for haemophilia A patients in Bulgaria.[17,18] If we consider patients to be ‘highly active’ up to the age of 40, then treatment with a prophylactic regime will decrease joint damage and better the quality of life without altering the cost-effectiveness of the treatment.[15] The cost of factor VIII is what influences the efficiency. Thus, any change regarding medicines cost would lead to a significant expense cuts and improvement of treatment efficiency.

The present analysis is limited in certain aspects. For example, it does not compare the benefits in regard to age. This, however, should not influence the overall data ratios, as the analysis encompasses all comparable regimes.[5,19] The study is done under conservative assumptions regarding the benefits of the prophylactic regime, since it also does not compare the increase in the number of haemorrhages with regard to age and does not include the expenses for prosthetic joints for patients who are treated with the on-demand regimen. If those expenses were to be considered, the prophylactic regime may prove to be cost-effective in comparison with the currently practiced regime of transition therapy after the age of 18.[20]

## Conclusions

The prophylactic therapeutic treatment of severe haemophilia A was found to decrease the costs of hospitalizations, surgical interventions and absences due to temporary incapacitation, as well as increase the quality of life of haemophilia patients. The application of the prophylactic regime for patients to 40 years of age would provide better treatment, increase the quality of life and decrease the incremental costs. 
